# Biological Monitoring of Healthcare Workers Exposed to Antineoplastic Drugs: Urinary Assessment of Cyclophosphamide and Ifosfamide

**Published:** 2018

**Authors:** Shadi Baniasadi, Maryam Alehashem, Masud Yunesian, Noushin Rastkari

**Affiliations:** a *Tracheal Diseases Research Center, National Research Institute of Tuberculosis and Lung Diseases (NRITLD), Shahid Beheshti University of Medical Sciences, Tehran, Iran. *; b *Department of Environmental Health Engineering, School of Public Health, Tehran University of Medical Sciences, Tehran, Iran. *; c *Center for Air Pollution Research (CAPR), Institute for Environmental Research (IER), Tehran University of Medical Sciences, Tehran, Iran.*

**Keywords:** Antineoplastic drugs, Cyclophosphamide, Healthcare worker, Ifosfamide, Occupational exposure, Oncology

## Abstract

Exposure of health care workers to antineoplastic drugs and subsequent adverse health effects is still an open issue. Very little has been studied on the extent of occupational exposure and handling conditions of antineoplastic drugs in Iran. We aimed to determine cyclophosphamide and ifosfamide concentrations in the urine samples of oncology healthcare workers. In addition, we assessed workplace safety controls that are important to decrease occupational exposure. Urinary samples of subject and control groups were collected to measure pre and post-shift cyclophosphamide and ifosfamide concentrations. Prior to sample collection, an occupational toxicologist observed and recorded working safety conditions for the healthcare workers during an eight-week period. Heath care workers were also asked about occurrence of acute adverse health effects. A total number of 425 chemotherapeutic drugs (389.83 g) were prepared during the study. Cyclophosphamide was detected in five pre-shift and nine post-shift urine samples. One pre-shift and four post-shift urine samples were positive for Ifosfamide. The urine samples of control group had no detectable concentrations of cyclophosphamide and ifosfamide. Personal protective equipment usage was not adequate for handling activities. Some adverse health effects reported by oncology personnel confirmed exposure to antineoplastic drugs. High percentage of oncology personnel was exposed to antineoplastic drugs that could be related to the large amount of drug preparations and inadequate safety controls. We recommend training of oncology personnel, implementation of safety controls, and periodic surveillance in order to minimize workplace contamination and occupational exposure to antineoplastic drugs.

## Introduction

Occupational exposure to antineoplastic drugs is still a matter of concern among healthcare workers who handle these drugs or work in contaminated area (1)_. _Several studies reported an increasing risk of leukemia, breast, and rectal cancer, premature delivery, and low birth weight in nurses potentially exposed to antineoplastic drugs. A number of biological monitoring studies also revealed that these exposures may result in genotoxic effects in pharmacists and nurses 

(2-4). 

Different professional organization such as Occupational Safety and Health Administration (OSHA) (5), National Institute for Occupational Safety and Health (NIOSH) (6), American Society of Health-System Pharmacists (ASHP) (7), and Oncology Nursing Society (ONS) (8) have issued several safe handling guidelines to minimize occupational exposure of healthcare workers. However, different levels of exposure have been reported by measuring surface contaminations (9-11), air contaminations (12, 13), urine drug concentrations (14-16), and genetic damages (17, 18). Detection of antineoplastic drugs in urine samples of workers shows their exposure through inhalation, dermal, hand-to-mouth, and accidental contacts. 

Our previous finding revealed that oncology healthcare workers experienced adverse health effects due to inadequate and ineffective safety controls (19). A biological monitoring is necessary in order to prove occupational exposure in our oncology setting. Consequently, we designed current study to determine cyclophosphamide and ifosfamide concentrations, two widely used antineoplastic alkylating agents, in the urine samples of our personnel. Additionally, we assessed workplace safety controls that are essential to protect healthcare workers against occupational exposure.

## Experimental

*Subjects*

Exposure to cyclophosphamide and ifosfamide was assessed in an oncology setting of a tertiary care teaching hospital. Healthcare workers (nurses, nurse assistants, cleaners, and secretor) who were potentially exposed to antineoplastic drugs were included. Non-exposed personnel from another ward of the hospital matched as a control group. Prior to sample collection, an occupational toxicologist observed and recorded conditions of working (preparation, administration, cleaning, and waste disposal) in terms of safety for eight weeks. Moreover, demographic profile, medical history, and laboratory tests results of the personnel, dose and number of prepared antineoplastic drugs, accidental contacts, and adverse health effects experienced by the personnel were documented using a questionnaire. Ethical permission for the study was obtained from the ethics review board of the National Research Institute of Tuberculosis and Lung Diseases.

*Urine sampling and analysis*

Urine samples were collected in 50 mL falcon tube before the start and at the end of the work shift. Because of cyclophosphamide and ifosfamide plasma half-life (~ 5 and 7 h), end of a 6 h work shift is appropriate time for sampling. On the other hand, the half-life of urinary excretion is 12-24 h for both drugs and pre-shift samples could reflect the extent of exposure over the previous day (1). The samples were stored at -20 °C until analysis. For detection of cyclophosphamide and ifosfamide, an aliquot of 5 mL urine for each sample was adjusted to pH 7, and 100 µL of 0.5 µg mL^-1^ TP aqueous solution was added, mixed, and extracted three times with 10 mL ethyl acetate. The organic layers were combined and evaporated to dryness under a gentle nitrogen stream. The residues were dissolved in 100 µL of ethyl acetate and derivatized by adding 100 µL of trifluoroacetic anhydride. After 20 min at 70 °C the reaction was stopped by evaporation to dryness. The residue was dissolved in 100 µL of isooctane; the solution was shaken vigorously using vortex-mixer for 1 min. Finally, 2 µL of the resulting solution was injected into the GC/MS. The analysis was performed on a GC/MS Agilent 6890 plus gas chromatograph equipped with a 5973 quadrupole mass spectrometer detector (Agilent Technologies, Palo Alto, CA, USA). The gas chromatograph was fitted with a DB-5 ms capillary column (30 m, 0.25 mm ID, 0.25 mm film thickness). The inlet was operated in splitless mode. The instrumental temperatures were set as follows: injector temperature 250 °C; initial oven temperature 70 °C, held for 1 min, increased to 250 °C at a rate of 15 °C min^-1^, held for 3 min and finally increased to 300 °C at a rate of 30 °C min^-1^, held for 5 min. The temperature of the transfer line was maintained at 300 °C. Helium was used as carrier gas at 1 mL min^-1^ (constant flow). 

The source and quadrupole temperatures were kept at 230 and 150 °C, respectively. The electronic beam energy of the mass spectrometer was set at 70 eV. Qualification was performed by comparing the acquired mass spectra and retention times to reference spectra and retention times which were acquired by injection calibration standards under identical GC/MS conditions. 

The compounds were quantified using selected ion monitoring (SIM) mode. The lower limit of quantification was set at 0.04 ng/mL for cyclophosphamide and 0.05 ng/mL for ifosfamide (20).

*Statistical analysis*

SPSS Software version 21.0 was used for data analysis. Descriptive statistics were applied to analyze the data related to demographic, safety controls, urine analysis, and adverse health effects. Normally distributed variables were presented as the mean ± SD. Statistical significance was considered when *P* < 0.05.

## Results

A total number of 60 urine samples were collected from the subjects and controls. Table 1 shows demographic characteristics of the participants. Ifosfamide, gemcitabine, cyclophosphamide were the three of the most frequently prepared and administered medications. During the study, a total number of 425 chemotherapeutic drugs (389.83 g) were prepared through 77 preparations. Gloves and mask were used by almost 100% of the personnel for preparation, cleaning, biohazard waste container replacement, and waste disposal. Whereas, 22.43% and 36.45% of the administrations were performed without gloves and mask, respectively. Percentages of personal protective equipment usage for different handling activities are presented in Table 2.

**Table 1:** Demographic characteristics of the participants

Characteristics	Subject	Control
**Number of participants (n)**	15	15
**Age (Mean ± SD)**	31.13 ± 6.45	37 ± 6.16
**Gender (Male/Female)**	6/9	5/10
**Job Experiences in oncology (Year) [Median (Range)]**	1 (0.25-11)	0
**Type of Job**		
**Nurse (n)** **Nurse-assistant (n)** **Cleaner (n)** **Secretor (n)**	9 3 2 1	9 3 2 1

**Table 2:** Percentage of personal protective equipment (PPE) usage for different handling activities

**PPE**	**Percentage of PPE usage for handling activities**				
	**Preparation**	**Administration**	**Cleaning**	**Replacement of biohazard waste container**	**Waste disposal**	
Gloves	100	77.57	100	100	100	
Mask	98.70	63.55	100	100	100	
Goggles	0	0	11.50	0	0	
Others (gown, hair and shoe cover)	0	0	0	0	0	

**Figure 1:**
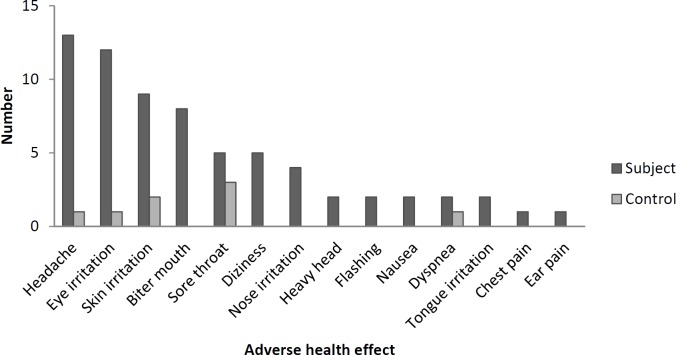
The total number of experienced adverse health effects in subject and control groups. The adverse health effects were asked through a questionnaire during eight-week period of the study (before sampling).

Figure 1 shows the number of experienced adverse health effects in the subject and control groups. Headache was the most frequent adverse effects reported by oncology healthcare workers. Antineoplastic drugs were often prepared in the first and middle of the work shift and most of the personnel (86%) experienced the symptoms in the middle of the work shift. Pre and post-shift urine concentrations of cyclophosphamide and ifosfamide are shown in Table 3. Cyclophosphamide was detected in five pre-shift and nine post-shift urine samples. One pre-shift and four post-shift urine samples were positive for Ifosfamide. The urine samples of control group had no detectable concentrations of cyclophosphamide and ifosfamide. One accident (skin contact with patient’s blood and drug, leakage and spilling of the drug) per week was observed within the study period. Risky behaviors (smoking, eating, drinking, resting, and storing food) were not performed by healthcare workers. Medical history and laboratory tests did not show any acute and chronic diseases among the workers (data not shown).

**Table 3:** Pre and post-shift concentrations of cyclophosphamide (Cyclo) and ifosphamide (Ifo) in the urine samples of oncology personnel

	**Job title**	**Handling activities**	**Job experience** ** (year)**	**Cyclo-Pre**	**Cyclo-Post**	**Ifo-Pre**	**Ifo-Post**
1	Nurse	no handling activity	11.00	ND*	ND	ND	ND
2	Nurse	Preparation and Administration	0.50	ND	ND	ND	ND
3	Nurse		0.16	ND	0.95	ND	0.21
4	Nurse		0.25	0.19	0.41	ND	ND
5	Nurse		0.50	0.21	0.85	0.12	0.35
6	Nurse		12.00	0.11	0.57	ND	0.12
7	Nurse	Administration	0.58	ND	0.22	ND	ND
8	Nurse		0.50	ND	0.48	ND	ND
9	Nurse		0.50	0.15	0.73	ND	ND
10	Nurse assistant		2.00	ND	ND	ND	ND
11	Nurse assistant		2.00	ND	ND	ND	ND
12	Nurse assistant		3.00	ND	0.51	ND	ND
13	cleaner	Cleaning and Replacement of biohazard waste container and waste disposal	3.00	ND	ND	ND	ND
14	cleaner		1.00	ND	ND	ND	ND
15	secretary	no handling activity	3.00	0.25	1.04	ND	0.24

* Non-Detectable.

## Discussion

Current study is the first one in Iran that determines the extent of exposure of healthcare workers to antineoplastic drugs by urine sample analysis. The results indicated 46.66% and 16.66% of the subjects′ urine samples were positive for cyclophosphamide and ifosphamide, respectively. Detectable amount of cyclophosphamide and ifosphamide in the urine samples of the subject group and negative result of the controls indicate occupational exposure to antineoplastic drugs in our oncology ward. Current findings are in accordance with a study that revealed 40% of the urine samples had detectable levels of cyclophosphamide (21). However, some studies did not find any evidence of trace amounts of cyclophosphamide and ifosfamide in the urine samples of healthcare personnel (1, 13). In line with our results, Connor *et al.* reported detectable amount of antineoplastic agents in the urine samples of oncology personnel who were not involved in handling of antineoplastic drugs (12). 

Occupational exposures to antineoplastic drugs have been studied through the determination of these agents in the surface, glove, air, and biological samples of healthcare workers (9-16). Several bio-monitoring methods have been developed to assess hazardous drugs in the biological samples (18, 22). Urine as easily accessible sample has been widely used in order to detect the extent of occupational exposure of oncology personnel to these drugs. However, the time of sampling should be considered for interpretation. Our result showed the presence of cyclophosphamide and ifosfamide in 33.32% and 6.66% of pre-shift samples, respectively, indicating high level of exposure during the previous working day. Large amount of cyclophosphamide [0.57 ng/mL (0.22-1.04)] and ifosfamide [0.26 ng/mL (0.12-0.35)] in post-shift urine samples also revealed unexpected exposure of the personnel to the drugs. 

Some adverse health effects reported by the subjects could also confirm occupational exposure to antineoplastic drugs. These symptoms rarely experienced by control group. Our findings showed that most reactions occurred in the middle of the work shift and lasted to the end or beyond of the shift. Krstev *et al.* also mentioned extension of the symptoms beyond the work shift (23). Since dermal contact and inhalation are the main ways of exposure to hazardous drugs (24, 25), preparation of antineoplastic dugs inside of a biological safety cabinet (BSC) and using suitable protection for skin and respiratory system could decrease occupational exposure. Assessment of workplace safety controls in oncology ward revealed that all preparations were performed in special room (preparation room) and inside of a BSC. Pethran *et al.* also showed detectable levels of cyclophosphamide in 7-40% of urine samples while laminar flow cabinets were used for the preparations (20). Based on the NIOSH guideline in safe handling of hazardous drugs, a well-functioning ventilation hood for preparation of antineoplastic agents could protect healthcare workers against occupational exposure. However, periodically evaluation of hood performance, that didn’t perform in our oncology ward, is essential to make sure of appropriate ventilation (6, 26). Costantinidis *et al.* found that improper location of BSC (between the window and entrance) could result in spreading antineoplastic aerosol to the ward environment (27). The same condition in our setting may be another reason for occupational exposure and detection of the drugs in personnel urine samples. Our oncology healthcare workers wore gloves and mask for all handling activities except administration. This finding reveals misconception of healthcare workers in terms of safety controls for administration of antineoplastic drugs (28). Incomplete wearing of personal protective equipment by our personnel was comparable with the other studies (27, 29). Goggles were rarely worn and some personal protective equipment such as gown, hair and shoe cover were not available in the ward. It has been shown that the lack of knowledge is an important reason for incomplete wearing of personal protective equipment by healthcare worker handling antineoplastic drugs (27, 30-32). Budget deficit and unqualified personal protective equipment for protection against hazardous drugs are also important limitations in developing countries. 

The limitation of our study is restricted number of healthcare workers that is due to urinary assessment of cyclophosphamide and ifosfamide in one oncology setting at a tertiary care center. The results of the current study will be used to conduct the future study in several oncology settings. 

## Conclusion

Current study showed relatively large amounts of antineoplastic drugs in the urine samples of our oncology personnel. They also reported some adverse health effects related to antineoplastic drugs exposure. We recommend training of oncology personnel, implementation of safety controls, and periodic surveillance in order to decrease occupational exposure to antineoplastic drugs.
